# Spatial heterogeneity of the relationships between environmental characteristics and active commuting: towards a locally varying social ecological model

**DOI:** 10.1186/s12942-015-0002-z

**Published:** 2015-03-25

**Authors:** Thierry Feuillet, Hélène Charreire, Mehdi Menai, Paul Salze, Chantal Simon, Julien Dugas, Serge Hercberg, Valentina A Andreeva, Christophe Enaux, Christiane Weber, Jean-Michel Oppert

**Affiliations:** University of Paris 13, Equipe de Recherche en Epidémiologie Nutritionnelle (EREN), UMR U1153 Inserm/U1125, Centre de Recherche en Epidémiologie et Biostatistiques Sorbonne, Paris Cité, Bobigny, France; University of Paris Est, Lab’Urba, Urban Institute of Paris, UPEC, Créteil, France; University of Strasbourg, Laboratoire « Image Ville Environnement » UMR 7362 CNRS, Strasbourg, France; CARMEN, Institut National de la Santé et de la Recherche Médicale U1060, University of Lyon 1, Institut National de la Recherche Agronomique U1235, CRNH Rhône-Alpes, Lyon, France; Service de Nutrition GH Pitié-Salpêtrière (AP-HP), Pierre and Marie Curie University, Institut Cardiométabolisme et Nutrition (ICAN), Paris, France

**Keywords:** Walking, Cycling, Active transportation, Geographically weighted regression, Spatial non-stationarity

## Abstract

**Background:**

According to the social ecological model of health-related behaviors, it is now well accepted that environmental factors influence habitual physical activity. Most previous studies on physical activity determinants have assumed spatial homogeneity across the study area, i.e. that the association between the environment and physical activity is the same whatever the location. The main novelty of our study was to explore geographical variation in the relationships between active commuting (walking and cycling to/from work) and residential environmental characteristics.

**Methods:**

4,164 adults from the ongoing Nutrinet-Santé web-cohort, residing in and around Paris, France, were studied using a geographically weighted Poisson regression (GWPR) model. Objective environmental variables, including both the built and the socio-economic characteristics around the place of residence of individuals, were assessed by GIS-based measures. Perceived environmental factors (index including safety, aesthetics, and pollution) were reported by questionnaires.

**Results:**

Our results show that the influence of the overall neighborhood environment appeared to be more pronounced in the suburban southern part of the study area (Val-de-Marne) compared to Paris inner city, whereas more complex patterns were found elsewhere. Active commuting was positively associated with the built environment only in the southern and northeastern parts of the study area, whereas positive associations with the socio-economic environment were found only in some specific locations in the southern and northern parts of the study area. Similar local variations were observed for the perceived environmental variables.

**Conclusions:**

These results suggest that: (i) when applied to active commuting, the social ecological conceptual framework should be locally nuanced, and (ii) local rather than global targeting of public health policies might be more efficient in promoting active commuting.

## Background

Promoting active transportation (walking and cycling) has become a priority in public health policies. The practice of daily active transportation has been shown to provide significant economic and health benefits. It contributes substantially to improving household budgets (reducing car-related expenditure [1]), limiting gas emissions, and decreasing other negative externalities, e.g. congestion, noise and pollution [2]. In addition, active transportation contributes to overall physical activity, which has been shown to have a protective effect against major chronic diseases (cardiovascular disease, type 2 diabetes, and certain cancers, according to the Physical Activity Guidelines for Americans [3]). However, active transportation represents only a small proportion of daily commuting. In France in 2008, 23% of commuting was by walking and 2.7% by cycling [4]. In the US, the corresponding figures were 10.5% and 1% in 2009, according to the National Household Travel Survey [5].

One major concern in developing relevant policies is the need for a better understanding of walking and cycling, which are complex behaviors, and their multiple determinants. Based on the socio-ecological conceptual framework, interacting factors include those related to the individual-level sphere and those associated with the social and physical environment [6,7]. In turn, social and physical environmental factors comprise both perceived and objective dimensions [8]. In this paper, a geographical approach was used to identify spatial variations in the associations between environmental characteristics and active transportation. Thus, the study could aid the development of more focused or locally adapted public policies and planning choices. For the purpose of this research, data on active commuting and explanatory factors were collected from a large sample of French adults and an innovative local-targeted regression technique was applied.

Associations between active commuting behaviors and social support (e.g. [9,10]), different physical environment dimensions, e.g. walkability and bikeability, land use, public transportation availability, safety, aesthetics, etc., in residential and/or work neighborhoods are documented in the literature (e.g. [11-24]). However, previous studies have largely been based on the implied and strong assumption that the relationship between individual/environmental factors and active commuting is spatially homogeneous, i.e. that pertinent factors operate in a similar manner everywhere. This is a necessary condition for the use of global regression models. Yet, non-stationarity, referring to the variation in relationships across space [25,26], is a very common phenomenon in any geographical dataset like place-level factors. For instance, a global positive association can be found between walking to work and overall walkability, but is this really the case everywhere throughout a city or a neighborhood?

In recent years, an innovative local-based regression technique has been gaining popularity for exploring spatial non-stationarity among data: the Geographically Weighted Regression model (GWR, [25]). GWR allows the parameter estimates to vary locally, unlike in global models where they remain constant. GWR fits local regression at each location by applying a weighting scheme (based on a kernel function) which gives more weight to neighboring locations [27,28]. The results emphasize the spatial patterning of relationships. The GWR technique has been successfully applied in a few health-related studies [29-37]. For instance, Chen and Truong [34] used GWR to highlight that township disadvantages increased obesity prevalence only in certain areas in Taiwan. Similarly, Chalkias et al. [37] showed that only certain zones in Athens (Greece) constituted an obesogenic environment.

To our knowledge, GWR has not yet been applied to explore the potential non-stationarity of environmental correlates of active commuting. The main objective of our study was thus to investigate whether several objective and perceived environmental determinants of active commuting behaviors varied across space, *ceteris paribus* (i.e. after adjusting for individual characteristics), in Paris and its immediate suburbs. The underlying idea was to question the relevance of using a general conceptual framework, such as the socio-ecological model, when analyzing spatial data including potential non-stationary processes. The study was thus positioned to raise questions about the potential gain of a more geographically nuanced theoretical model, taking into account area-specific attitudes and behaviors. To explore this issue, a multivariate geographically weighted Poisson regression (GWPR) model was used, based on data from French adults.

## Methods

### Study area and population

Information about active commuting behaviors and some of the explanatory factors was derived from the Nutrinet-Santé study, an ongoing web-based cohort launched in France in May 2009, which focuses on relationships between nutrition and health [38]. Briefly, participants aged 18 y. or older completed a set of questionnaires assessing demographic and socio-economic characteristics, as well as physical activity and perceived residential environment (response rate of 48.5%). Residential addresses were obtained from all participants, geocoded to the parcel or street levels and implemented as a shapefile in a geographical information system (GIS). This study was conducted according to the guidelines laid down in the Declaration of Helsinki, and all procedures were approved by the Institutional Review Board of the French Institute for Health and Medical Research (IRB Inserm n° 0000388FWA00005831) and the *Commission Nationale Informatique et Libertés* (CNIL n° 908450 and n° 909216). All participants gave their written electronic informed consent to take part in the study.

**Figure 1:**
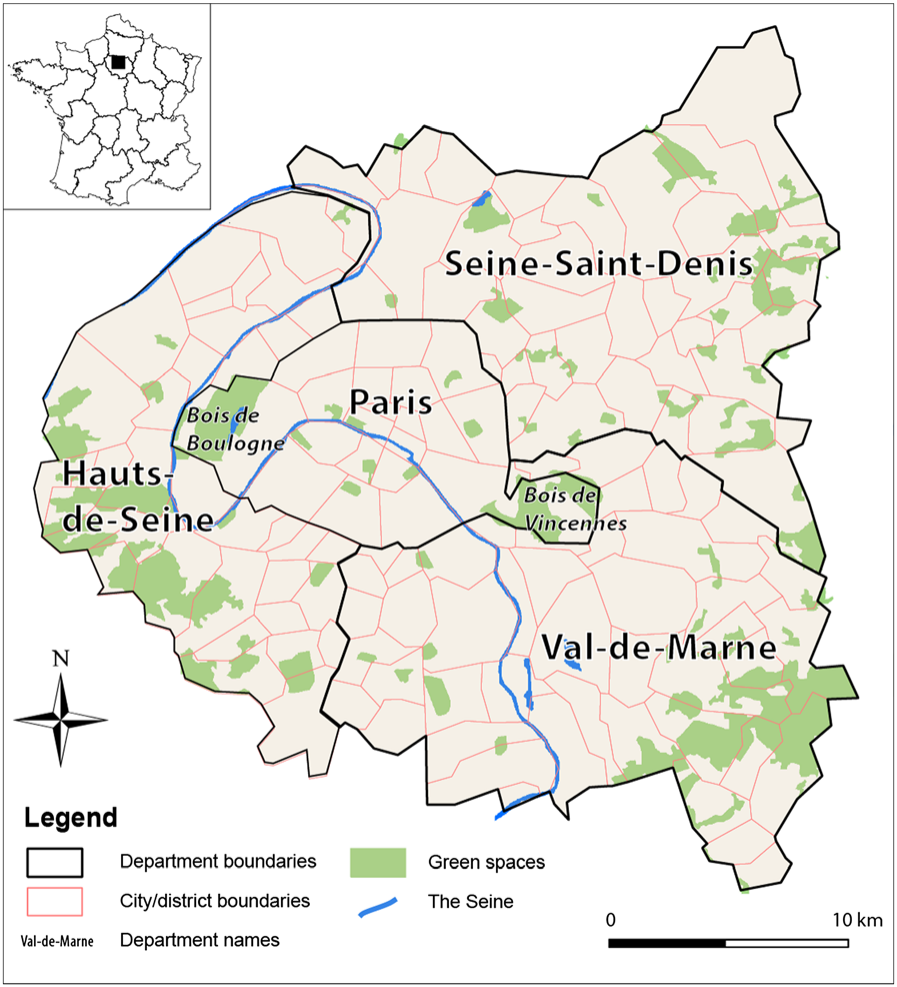
**Location map of the study area showing Paris and its three immediate suburbs.**

The area covering Paris and its three surrounding *départements* called the “Petite Couronne” (Figure 1), was targeted because it is the most populated region in France, giving access to a large sample of participants. Indeed, this urban area sprawls over 762 km^2^ and has more than 6.6 million inhabitants (2010 French Census) with a population density of approximately 8,700 hab./km^2^. More than 2.2 million individuals live in the city of Paris alone, leading to a population density of 21,347 hab./km^2^.

### Outcome variable: active commuting (walking and cycling to and from work)

Participants reported their time spent on active commuting. For each subject, the variable used was the mean of the hours spent walking and biking to/from work per week during the past 4 weeks.

### Explanatory variables

#### Environmental variables

A set of environmental variables was assessed to characterize the residential neighborhood of each individual. Variables were categorized as objective (i.e. GIS-based) or perceived (i.e. reported via a questionnaire).

#### Objective variables obtained from GIS (built and social)

**Figure 2:**
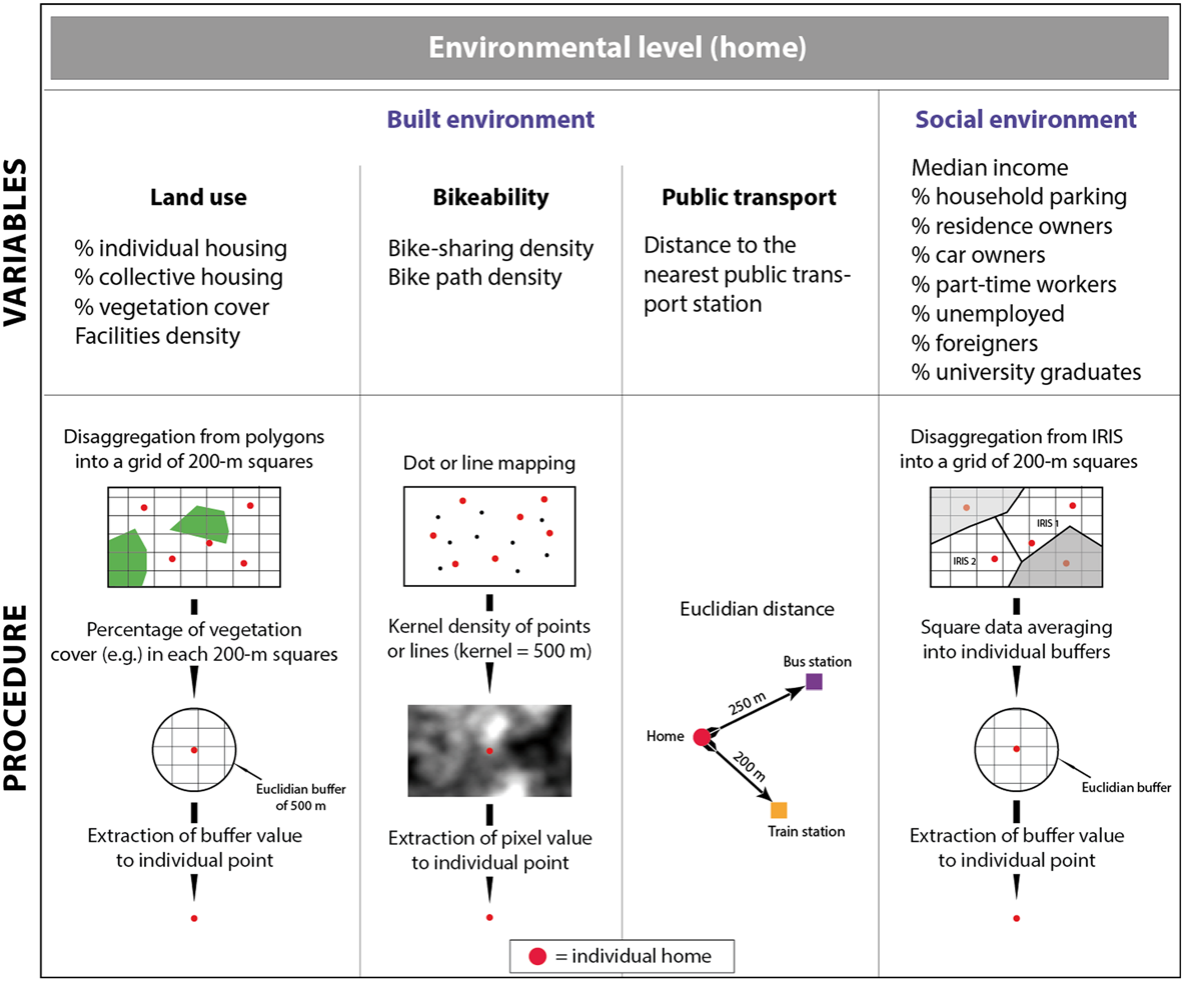
**GIS-based schematic procedure for the calculation of objective (both built and social) environmental variables.**

Overall, fifteen GIS-based variables were obtained from different sources. They were related to either the built (7 variables) or the social (8 variables) environment (Figure 2). The GIS procedure used for calculating each variable is illustrated in Figure 2. All the geoprocessing steps were performed with ArcGIS 10.1 (ESRI Inc., Redlands, CA, USA).

The built environment set encompassed 7 variables, representing 3 distinct groups (land use and facilities, level of bikeability, and availability of public transportation). These were chosen because they have been shown to be associated with transportation-related physical activity [39-41]. According to this literature, expected relationships between walking for transportation and proximity facilities and land use mix are positive, even if the specifics of these associations are less clear.

(i) Land use and facilities

Land use included the percentage of area covered by individual housing, collective housing and vegetation, respectively, as well as proximity facilities. Data on housing and vegetation were provided as shapefiles (polygons) by the Paris Region Urban Planning & Development Agency (IAU Île-de-France, 2008, scale: 1:5000). Data on proximity facilities were given as the number of 26 different types of facilities, e.g. banks, bakeries, drugstores, restaurants, etc., by the IRIS Census unit. IRIS areas (acronym for “Aggregated Units for Statistical Information”), provided by the French National Institute of Statistics and Economic Studies (INSEE, www.insee.fr), represent the smallest unit for dissemination of French infra-municipal data. They include an average of 2000 residents per unit and are homogeneous in terms of housing and socio-economic conditions. Data on land use and facilities were then disaggregated into a grid of 200 × 200 m cells, in order to obtain a spatially homogeneous data net. Finally, these data were linked to each individual within a Euclidian buffer of 500 m around the residential address (mean values of the cells included in the buffer), as this distance is commonly used in accessibility studies [42,43].

(ii) Level of bikeability

Bikeability (positive expected association with active commuting), including bike-sharing facilities and bike path densities, was assessed by fixed kernel density estimation (KDE) with a bandwidth of 500 m, which is equivalent to a Euclidian buffer for polygon-shaped variables. KDE is a smoothing geostatistical technique to transform a point or a line pattern into a continuous surface map of density (raster), with an estimated value for each cell. This method has been extensively used in other accessibility studies (e.g. [31,44-46]). Bike-sharing accessibility was assessed by calculating kernel density estimation on the 1,230 bicycle stations of the Parisian bicycle-sharing system, called “Vélib”. Next, the value of the overlapping density raster cell (for bike-sharing and bike path densities) was assigned to each individual residential address.

(iii) Public transportation availability

Public transportation availability (positive expected association with active commuting) was assessed by calculating the distance to the nearest subway, bus or train station (provided by IAU) from each individual home.

(iv) Socioeconomic environment

The socioeconomic level of the residential environment has been considered since it is expected to be positively associated with overall physical activity, including active commuting [47]. Eight neighborhood-level socioeconomic variables, from the Census database (www.insee.fr), were included and disaggregated into the 200-m square grid (following the same procedure as used for land use and facilities) (see Figure 2): percentage of foreign residents, unemployed, part-time workers, university graduates, homes occupied by their owners, car owners, households with a parking space, and median income.

#### Statistical analyses of objective environmental variables

The 7 physical and 8 socio-environmental variables were substantially multicollinear. As a valid regression analysis requires the explanatory variables to be independent, a suitable solution to this statistical issue is to transform the set of correlated variables into synthetic uncorrelated variables, named principal components (PC). In PC analysis (PCA), the principal components retained are those that explain the maximum amount of variance of the original data. This method was used in our study to avoid dropping any explanatory variable and to keep as much information as possible. PC were retained as new explanatory variables when eigenvalues were greater than one. PC coordinates were assigned to individuals and then mapped to facilitate the interpretation. The PCA was carried out with varimax rotation to represent the linear proximity among variables. It was conducted only with objective variables since we were interested in investigating separately the spatial variation in the influence of objective and perceived variables on active commuting.

The PCA results revealed that the first five principal components accounted for 84% of the total inertia of the original variables. The first two PC explained 64% of the variance, whereas the following three PC had an eigenvalue below 1. For this reason, only the two first PC were kept as independent variables in the main analysis. The highest eigenvectors for the first component (PC1) were associated with low rates of car ownership, individual housing, and households with a parking space, but with high rates of home ownership, collective housing, and high facility and bike-sharing densities (Table 1). This first synthetic variable was thus related to the built environment density. The highest eigenvectors for PC2 were associated with high median income, a high proportion of university graduates, and low proportions of foreign residents and unemployed. This second synthetic variable was related to the socio-economic environment (high values indicate well-to-do areas).

**Table 1:** **Results of the principal component analysis conducted with the fifteen objective environmental variables**

**Variable**	**Principal component 1 Densely built-up areas & facility availability**	**Principal component 2 Socio-economics: well-off neighborhoods**
Median income	−0.01	**0.51**
% households with a parking spot	**−0.36**	0.04
% home owners	**0.32**	0.08
% car owners	**−0.38**	0.04
% individual housing	**−0.33**	−0.06
% collective housing	**0.34**	0.13
% part-time workers	0.17	−0.21
% unemployed	0.11	**−0.49**
% foreign residents	0.17	**−0.42**
% university graduates	0.20	**0.44**
% vegetation cover	−0.20	0.17
Facility density	**0.30**	0.05
Distance to public transportation	−0.15	−0.03
Bike-sharing density	**0.34**	0.10
Bike path density	0.10	0.05
Overall KMO score = 0.78; Bartlett’s test p < 0.001		

Values in bold font are greater than |0.3|.

#### Perceived environment variables

Three perceived residential neighborhood variables were defined by specific questions in a self-administered questionnaire. These three questions were derived from the ALPHA questionnaire designed to measure the relationship between physical activity and the environment in a European context [48]. Briefly, the three questions were related to (i) bike safety in road traffic (‘cycling is unsafe because of the traffic’), (ii) pollution (‘there is too much pollution in my neighborhood’), and (iii) aesthetics (‘my neighborhood is not clean and not well-maintained’). Responses included five modalities based on a Likert-type scale (strongly agree, somewhat agree, neither agree nor disagree, somewhat disagree, strongly disagree). To account for substantial multicollinearity among these three variables, an index was built using principal component analysis. This index accounted for 48% of the total variance. The factor loadings were 0.72 for bike safety, 0.69 for pollution and −0.68 for aesthetics. Therefore, a high value in this synthetic index indicates individuals who reported a low level of pollution, a feeling of safety for cycling and a low level of aesthetics. A positive relationship between this synthetic variable and active commuting is expected [49].

#### Individual variables

Individual data included age, gender and education (divided into two categories, < high school, ≥ high school), number of motor vehicles per household, number of bikes per household, and an indicator related to the possession of a transit pass (yes or no). Finally, two work-related variables were added: self-reported commuting time (divided into tertiles) and availability of a parking space at the workplace (yes or no).

### Statistical modeling

Given the non-Gaussian, zero-inflated distribution of the outcome variables (walking and cycling to and from work), each value was rounded to the nearest half-unit (0.5). This procedure of discretization enabled the variable to be modeled with a Poisson regression. First, a global Poisson model (GPR) was carried out, i.e. parameter estimates were kept constant to explore global relationships between environmental variables and active commuting, while adjusting for individual variables. Secondly, a geographically weighted Poisson regression [28,30] was conducted to account for the possible spatial non-stationarity of these relationships. The statistical specifications of GPR are described in Appendix A.

### Local model (GWPR)

Geographically weighted regression aims to capture spatial non-stationarity, i.e. spatially varying relationships, in a regression model by allowing regression parameters *β*_0_, …, *β*_*k*_ to vary with location. To do this, GWPR incorporates spatial coordinates in the model. Since this study assumes that only environmental factors have spatial effects and not individual ones, we performed a semiparametric GWPR, i.e. we kept fixed the coefficients of the individual factors [30]. The resulting equation of the semiparametric GWPR we used is expressed as follows:

1 $$ \log\ {\lambda}_i={\displaystyle {\sum}_k{\beta}_k\left({u}_i,{v}_i\right){x}_{ik}+{\displaystyle {\sum}_m{\gamma}_m{x}_{im}}} $$

where *β*_*k*_(*u*_*i*_, *v*_*i*_) are local model parameters associated with environmental factors and specific to residential location of subject *i*, (*u*_*i*_, *v*_*i*_) denoting the coordinates of residential location of subject *i*, *x*_*ik*_ is the value of the *k*^th^ environmental variable at residential location of subject *i*, and *γ*_*m*_ are model parameters associated with the individual variables *x*_*im*_, not assumed to depend on geographical location.

**Figure 3:**
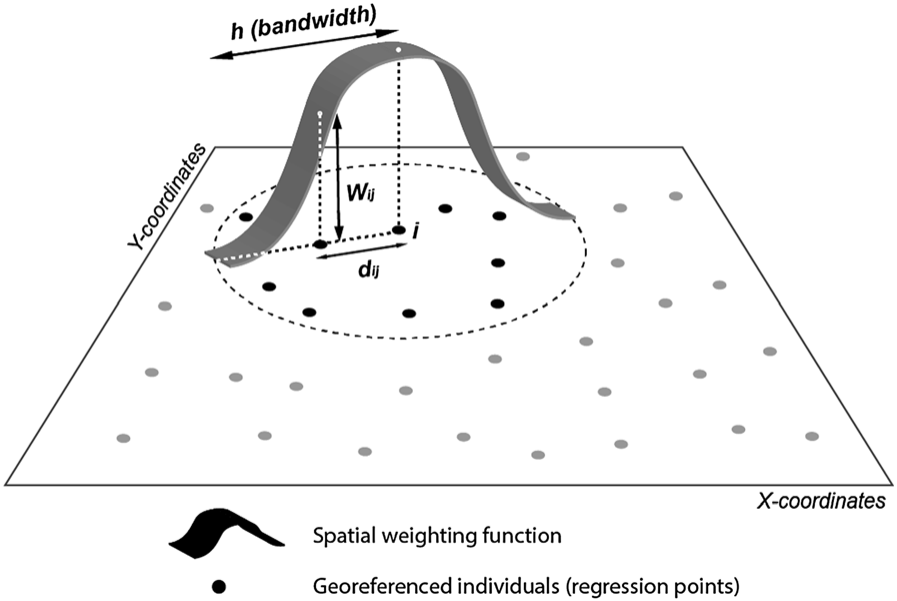
**Schematic representation of the geographically weighted regression and its spatial parameters.**

A key step in the development of GWPR consists of calibrating the model by a kernel regression method in order to estimate smoothed geographical variations in the parameters with a distance-based weighting scheme [30]. GWPR uses a spatial kernel since it is assumed that observations near point *i* have more influence on the estimation of parameter *β*_*k*_(*u*_*i*_, *v*_*i*_) than do observations located farther from *i*. In other words, GWPR integrates multiple local regressions within an overall framework, as illustrated in Figure 3. The estimation of the parameters is described in Appendix B. Next, parameters at location *i* are estimated by maximizing the geographically weighted log-likelihood [30]. Thereby, the geographical weight structure can be based on one of two types of kernel function, Gaussian or bi-square [25,28]. The kernel’s bandwidth can be set as fixed (based on metric distance) or adaptive (based on a constant number of neighbors considered in each regression calculation). Adaptive kernels are suitable when the units of analysis are irregularly distributed across space, which is the case here: respondents are sparse near the study area boundaries and absent from the two large green spaces of Paris (*Bois de Boulogne* and *Bois de Vincennes*, see Figure 1). For this reason, the adaptive kernel method was used, making sure that each local regression encompassed enough regression points irrespective of the location, coupled with a bi-square weighting scheme, which gave better results than the Gaussian one alone (see details in Appendix C).

The maximum number of neighbors in each regression model was determined by minimizing, in an iterative way, the corrected Akaike’s Information Criterion (AIC*c*), which is a statistic based on the log-likelihood of the model, weighted by the actual number of parameters [50]. In this study, the number of neighbors minimizing the AIC*c* was 800 (after testing down from 4,000 to 50, every 50). This amounted to an adaptive bandwidth size varying from 3,150 m to 19,250 m and a mean distance of 5,650 m, knowing that the study area spreads over approximately 30,000 m.

AIC*c* was also used to compare the performance of both global and local models. The model with the smallest AIC*c* should be selected as an optimal model called MAICE (minimum AIC estimator, see [30]. A difference in AIC*c* values of more than 2 is considered substantial.

Global models were performed with SAS 9.3 software (SAS Institute Inc., Cary, NC, USA) and the academic piece of software GWR 4.0 was used to calibrate and run geographically weighted models. This software is a tool for modelling varying relationships among variables by calibrating GWR and Geographically Weighted Generalized Linear Models (GWGLM) with their semi-parametric variants (https://geodacenter.asu.edu/gwr_software, see Nakaya et al. [51] and Nakaya [52] for additional details). Maps with continuous values were based on an interpolation procedure (inverse distance weighting) and were processed with ArcGIS 10.1 (ESRI Inc., Redlands, CA, USA). Finally, a measure of statistical significance (pseudo t-value) of GWPR estimates was added visually as isolines for each local term map. A value greater than |1.96| indicates a p-value < 0.05. Mapped GWPR estimates are log-odds, with negative and positive values meaning negative and positive relationships, respectively.

## Results

### Descriptive statistics

#### Characteristics of the study population

The participation rate for the questionnaire was 48.5%. 61.7% of the valid questionnaires were kept, resulting in a sample of 4,164 respondents. Table 2 shows that they are 43.6 years-old on average, 78% of them are women, 83% have an educational level above high school and approximately 46% have a transit pass. 41% of respondents do not practice active commuting, while approximately 95% walk or cycle less than 5 h/week. The mean time for active commuting among participants reporting any such activity (59%) is 2.3 h/week. Regarding the perception variables, around 47% of the respondents report too much pollution, approximately 40% report that biking is unsafe in their neighborhood, and 16% find that their neighborhood is not clean (Table 2).

**Table 2:** **Descriptive statistics of the variables used in the analysis**

**Variable (N = 4164)**	**Mean (or %)**	**Min**	**Max**	**STD**
***Active commuting (outcome)***				
% No	41	/	/	/
% Yes	59	/	/	/
*If yes (h/week)*	2.34	0.1	10.8	1.8
***Individual variables***				
Age	43.6	19	87	13.3
Gender				
*% Men*	22.0	/	/	/
*% Women*	78.0	/	/	/
Education				
*% < high school*	16.8	/	/	/
*% ≥ high school*	83.1	/	/	/
Parking at work				
*% Yes*	36.7	/	/	/
*% No*	63.3	/	/	/
Transit pass				
*% Yes*	45.8	/	/	/
*% No*	54.3	/	/	/
Commuting time				
*% 1st tertile*	36.2	/	/	/
*% 2nd tertile*	30.6	/	/	/
*% 3rd tertile*	33.1	/	/	/
Number of motor vehicles owned	1.03	0	8	0.95
Number of bikes owned	1.30	0	4	1.38
***GIS-based environmental variables***				
PC1 (densely built-up areas)	0	−8.6	5.3	2.5
PC2 (well-to-do areas)	0	−8.5	4.0	1.8
***Perceived environmental variables***		**% agree**	**% neither agree nor disagree**	**% disagree**
Too much pollution		47.3	24.6	28.1
Neighborhood is not clean		15.8	14.3	69.9
Biking is unsafe		34.4	25.7	39.9

#### Characteristics of the environment

**Figure 4:**
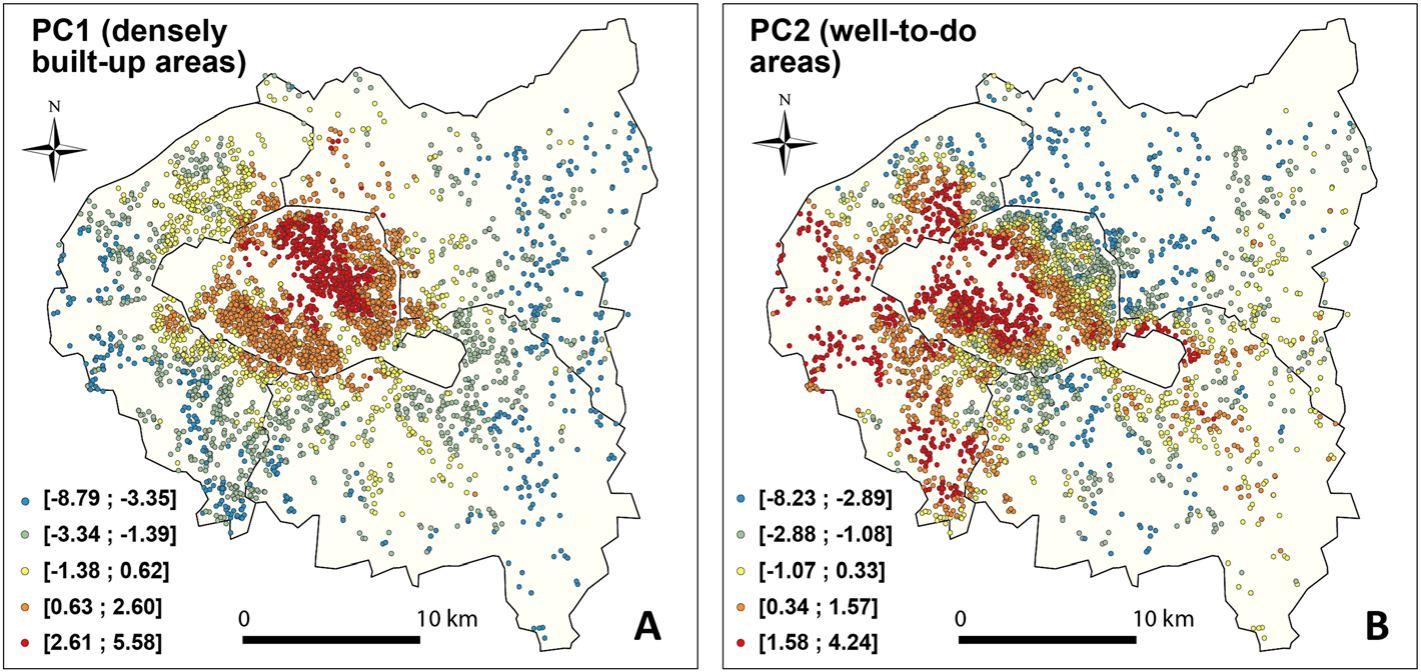
**Map view of the first two components derived from the principal component analysis and kept as explanatory variables. A**. The first component (PC1) refers to the built environment (densely built-up areas and facility density) and is characterized by high values in Paris. **B**. The second component (PC2) is related to the socio-economic environment (high values indicate well-to-do areas).

For the first principal component (PC1) related to the built environment density, the highest values are located within Paris and its immediate suburbs, as expected, whereas values tend to decrease further outwards (Figure 4A). For the second principal component (PC2) related to the socio-economic environment, the highest values are found in neighborhoods mostly encompassing the southern and western parts of Paris and the surrounding suburbs, as well as areas surrounding the *Bois de Vincennes* in the eastern part of the city (Figure 4B). The lowest values of PC2 are located in the northern part of the area, especially the western part of the Seine-Saint-Denis *département*.

#### Global relationships between active commuting and environmental variables

Total deviance explained by the global regression model is 24% with an AIC*c* of 6,733 (Table 3). Results of the parameter estimations via the global Poisson regression are summarized in Table 4. Nagelkerke’s R^2^ for the full model is 0.317 (meaning that approximately 32% of the total variance is explained by the model), with a value of 0.297 for the model including only individual-level variables, i.e. the individual level accounts for around 94% of the full model pseudo R^2^.

**Table 3:** **Overall performances of both global and local (bi-square kernel) regressions used for modeling associations between active commuting behaviors and individual and environmental factors**

**Model (N = 4164)**	**Deviance (D)**	**Effective number of parameters (k)**	**AIC** ***c*** (D + 2*k)	**Deviance explained**
GPR	6733	12	6757	0.24
GWPR (800 neighbors)	6207	167	6556	0.30

**Table 4:** **Parameter estimations from the global Poisson regression model**

	**Variable**	**Log-odds**	**OR**	**Wald 95% CI**		**p-value**
	Intercept	−0.10	0.91	0.68	1.21	0.498
Individual level	Age	0.00	1.00	1.00	1.01	0.085
	Gender (ref = male)	0.04	1.04	0.96	1.12	0.393
	Education (ref = < high school)	−0.12***	0.89	0.82	0.96	<.0001
	Parking at work (ref = yes)	−0.29***	0.75	0.69	0.80	<.0001
	Transit pass (ref = yes)	−0.05	0.95	0.88	1.03	0.148
	Commuting time (ref = 1st tertile)	0.67***	1.96	1.87	2.05	<.0001
	Number of vehicles owned	−0.19***	0.83	0.79	0.87	<.0001
	Number of bikes owned	0.09***	1.10	1.07	1.13	<.0001
Environmental level	PC1 (densely built-up areas)	0.05***	1.05	1.03	1.07	<.0001
	PC2 (well-to-do areas)	0.01	1.01	0.99	1.03	0.496
	Neighborhood perception (too much pollution and not clean) (0 = strongly agree; 5 = strongly disagree)	0.04**	1.04	1.02	1.06	0.001

***p < 0.001, **p < 0.01.

Regarding the environmental variables, after controlling for individual ones, the results show that active commuting is positively associated with the first principal component (OR = 1.05, 95% CI 1.03-1.07, implying that an increase of one unit of the densely-built level of the neighborhood is associated with an increase in active commuting), but no global relationship was detected with the second principal component (socio-economic level of the neighborhood). Finally, the perception of the neighborhood environment is significantly associated with active commuting (OR = 1.04, 95% CI 1.02-1.06).

#### Spatial variations in the relationships

Total deviance explained by the GWPR model is 30% with an AIC*c* of 6,556 (Table 3), which is a better goodness-of-fit diagnostic than the global regression model. Although the mean ORs are relatively close to those of the global regression (Table 5), the range of GWPR OR estimations shows the non-stationarity of the relationships between the environmental variables and active commuting in the study area.

**Table 5:** **Parameter estimations from the semiparametric geographically weighted Poisson regression model (after adjusting for individual variables)**

**Variable**	**Mean log-odds**	**Mean ORs**	**STD log-odds**	**Min ORs**	**Max ORs**	**Range ORs**
Intercept	0.01	1.01	0.12	0.75	1.43	0.68
PC1 (densely built-up areas)	0.03	1.03	0.06	0.84	1.25	0.41
PC2 (well-to-do areas)	−0.00	0.99	0.03	0.89	1.13	0.24
Neighborhood perception (too much pollution and not clean) (0 = strongly agree; 5 = strongly disagree)	0.03	1.03	0.06	0.85	1.27	0.42

**Figure 5:**
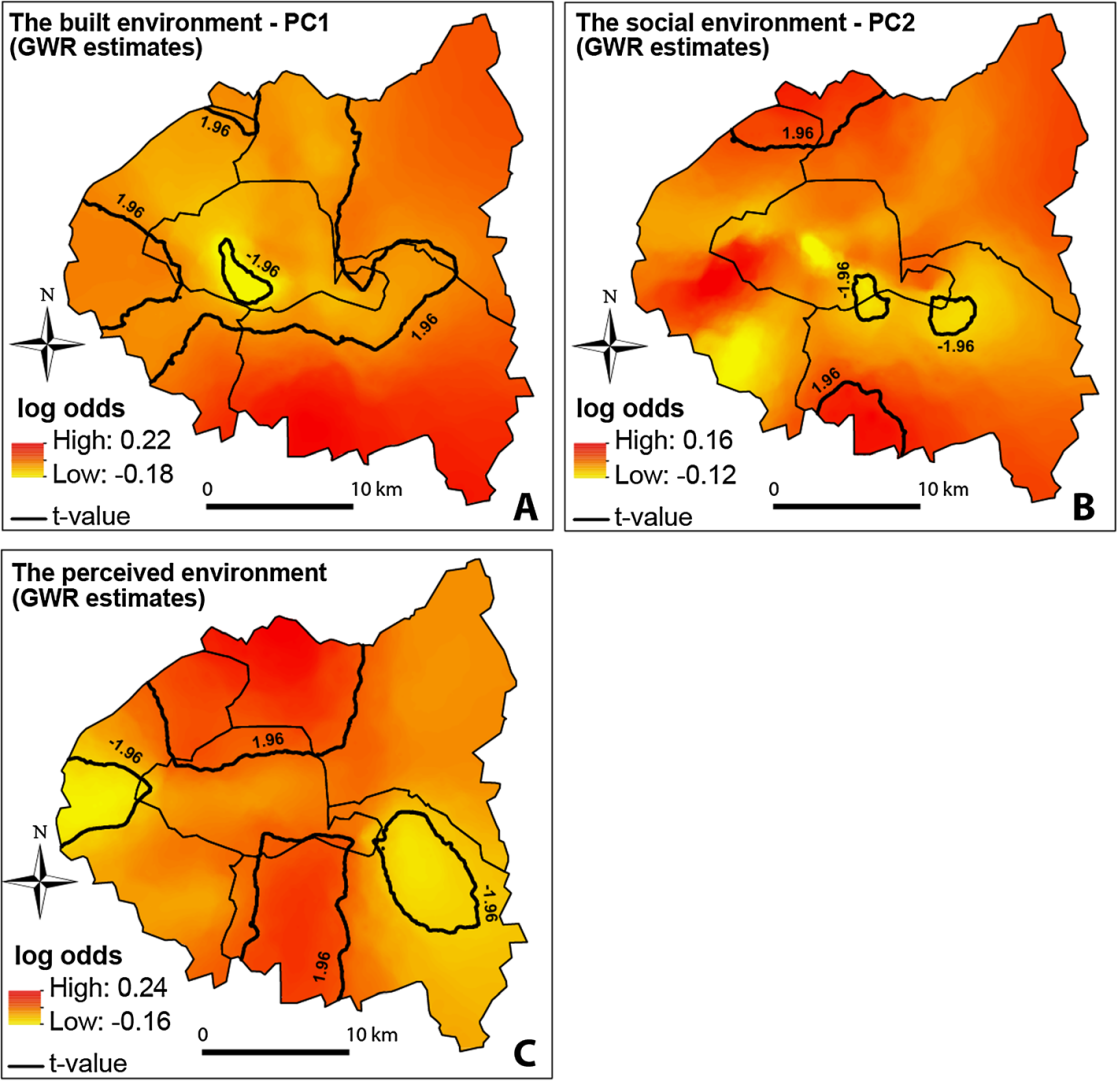
**Map results of the geographically weighted Poisson regression parameters (log odds) for the built (A), the social (B) and the perceived (C) environment.** Positive values of the log-odds (in red) indicate positive relationships between the respective explanatory variable and active commuting, and negative values of the log-odds (in yellow) indicate negative relationships. A pseudo t-value > |1.96| shows significant associations (p < 0.05).

For the three environmental variables, and while controlling for individual-level covariates, ORs are spread on both sides of 1, meaning that the relationships are sometimes negative, sometimes positive, and sometimes non-significant according to the location in the study area (Figure 5). Relationships between active commuting and the built environment vary substantially, with ORs ranging from 0.84 to 1.25 (Table 5). The relationships are significant and positive in the southern part (*département* of Val-de-Marne) and the northeastern part of the study area (Figure 5A). Elsewhere, the relationships are mostly non-significant, except at specific locations, such as in a small area in Paris where the relationships are negative. While the global model shows no significant associations between active commuting and the socio-economic environment, GWPR indicates some local nuances, as small parts of the area (extreme north and south) exhibit significantly positive ORs (>1.10, Table 5 and Figure 5B).

Perceived environmental variables also show some non-stationarity (Figure 5C). In northern and southern central parts of the area, associations are significantly positive, whereas they are non-significant elsewhere, or even negative in some parts of the Hauts-de-Seine and Val-de-Marne *départements.*

## Discussion

Using a GWPR, we have clearly demonstrated some spatial heterogeneity within the relationships across our study area. This represents the main novel result of our study. To the best of our knowledge, there is currently no other study on spatially varying relationships between the environment and walking or cycling for commuting purposes. We have shown that the associations of environmental factors with active commuting are area-specific. In other words, the relative influence of the specific environmental characteristics of the neighborhood (built, social and perceived) differs by location. For instance, the southern part of the study area (*département* of Val-de-Marne) is generally characterized by significant positive associations between environmental characteristics and active commuting. In contrast, in the major part of Paris or in the northern part of the Hauts-de-Seine *département*, environmental characteristics and perception of the neighborhood are not associated with walking and cycling for commuting purposes. Our results also show the complexity of the relationship between the environment and active commuting. At the same location, certain environmental variables may be associated with the outcome, while others may not.

Regarding the global relationships, we have highlighted that both perceived and objective environmental factors are significantly associated with active commuting, as expected. The importance of both the individual and the environmental level for active transportation is in line with existing evidence (e.g. [14,17,24,49,53-57]) based on the social ecological framework [8,58]. Although some environmental characteristics have significant relationships with active commuting, they only weakly contribute to explaining its total variance (as shown by the respective Nagelkerke’s pseudo R^2^ of the nested models (R^2^ = 0.30 without the environment level, and 0.32 with it)). This corroborates findings by Giles-Corti and Donovan [59] in Australia, and Ogilvie et al. in the UK [55]. For instance, Ogilvie et al. [55] highlighted that in urban neighborhoods of Glasgow, 18.7% of the total variance in active travel was explained by personal correlates, and 20.1% when environmental-level variables were added. These authors concluded that including environmental characteristics did not substantially modify the influence of the personal characteristics on the associations studied.

### Interpretation of the observed spatial non-stationarity

The interpretation of the spatial patterning of the relationships needs further and deeper investigation. Three potential causes of parametric instability of the regression parameters have been identified by Fotheringham et al. [28]:

(i) The non-stationarity could be due to random sampling variations and hence not related to any underlying spatial process.
(ii) The relationships might be intrinsically different across space, in other words “there are spatial variations in people’s attitudes or preferences or there are different administrative, political or other contextual issues that produce different responses to the same stimuli over space”.
(iii) The non-stationarity could also indicate that the model suffers from major misspecification or omission of key variables (or representation by an incorrect functional form).

The demarcation between the second and third potential causes of parametric instability may seem blurred in some cases. Can relationships really be intrinsically different across space? As discussed by Blainey [60], space may represent “merely a proxy for societal factors which are not captured by the model”. The crucial issue here is that such space-related societal factors are sometimes very difficult to define, and hence hard to quantify in a model.

However, in some instances, varying relationships seem to be driven both by the level of and the variance in the explanatory variable involved. For example, regarding the variable related to densely-built areas, the absence of a relationship in the major part of Paris could be explained by the fact that facility and building density is already extremely high in the city, so that one additional unit (of density or facility availability) would have a limited marginal effect on active commuting behavior. In contrast, in the less densely-built areas surrounding Paris (south and east), an increase of one unit regarding this variable would have a more noticeable impact. In that case, the instability of the parameters associated with the facility availability would only be due to a threshold effect of the variable itself and not directly linked to another contextual effect. The same rationale was used by Lu et al. [61] to explain the heterogeneity of relationships between the number of buildings and non-motorized traffic in Burlington, Vermont, USA. The number of buildings was associated with non-motorized traffic in the suburbs (with low building density), but not in the city center (with high building density).

Local associations with the perceived environment also show interesting spatial patterns. Unlike the objective environmental variables, the perceived ones do not follow any obvious spatial distribution (meaning that they are not correlated with objective measures). This means that spatial non-stationarity associated with these variables could be due to the omission of variables in each local context drawn through the GWPR estimates (Figure 5C). For instance, a local confounding factor may have been omitted from the model, leading to a spurious association. In some locations, reporting a low level of pollution and a strong feeling of safety for cycling is associated with a decrease in active commuting, which is counter-intuitive. We can hypothesize that in such places, these perceived neighborhood characteristics may be correlated with exposure to a limited traffic volume, while such little traffic may indicate a less walkable/bikeable environment in terms of infrastructure density [62].

Small single patterns of non-stationarity often remain difficult to interpret in detail, partly because the possibly omitted local variables are often not easily quantifiable, being the results of complex local interactions. In particular, the strength of social interactions within a place may lead to a homogenization of the relationships [63]. However, exploring these interactions, opening “the black boxes of places” [64], needs further and deeper quantitative and qualitative investigations. It is still advisable to keep an overview of the overall spatial patterning of non-stationarity, since this enables some boundaries between different local contexts to be drawn, wherein, for complex reasons, relationships converge.

### Implications of the findings

The non-stationarity of spatial datasets has been demonstrated in other studies dealing with health-related outcomes, such as obesity [37], cardiovascular mortality [34], and health care system organization [35]. Such observations lead to questions about the relevance of using global models, which tend to smooth the effects of one or another variable across the entire area, whereas these effects are in fact area-specific. Considering local models over global ones has potential implications for public health policies. As emphasized by Yang and Matthews [35], GWR-based analyses in health-related research could be used as a tool for place-specific targeting and/or tailoring of public health interventions. For example, potential interventions by local authorities regarding bike safety in traffic, e.g. installing separate bike paths away from roads, might have a stronger impact on cycling behaviors in the southern and northern parts of our study area than in the eastern part (Figure 5C). In addition, increasing bike-sharing stations and facility density (i.e. contributing to increasing PC1) would be more useful in the Val-de-Marne *département* than in the Hauts-de-Seine *département*, or in Paris (Figure 5A), in terms of active commuting. Such place-level prioritizing could be not only more efficient than whole-area interventions for promoting active transportation, but could also ensure substantial cost-efficiency in planning policies.

### Theoretical considerations: toward a locally varying social ecological model

According to Sallis et al. [58], the first principle of ecological models is that multiple levels of factors, including individual and environmental ones, influence health behaviors. Our results on active commuting behaviors, namely the spatial variability of the relative influence of individual and environmental factors, suggest the importance of considering the local context. In other words, the social ecological framework needs to be locally adapted, according to the spatial patterning of the relationships. In certain areas, policy-makers might achieve better results by acting on one particular level rather than on multiple ones. This also suggests expanding the fourth principle proposed by Sallis et al. [58]: ecological models are most efficient when they are not only *behavior*-specific, but also *area*-specific.

Beyond the physical activity domain, the idea of spatially varying relationships also fits the theoretical framework developed by Lytle [65] for eating behaviors. This author hypothesized that the relative influence of individual, environmental and social factors on the proportion of variance explained in eating behaviors varied as a function of the level of restriction of the environment, specifically: “the more restricted an environment is with regard to availability and accessibility of healthy, inexpensive options, the more influence the physical environment may have with regard to food choices that are made” [65]. Our GWPR analyses provide empirical evidence for the application of Lytle’s assumption to active commuting behaviors.

### Strengths and limitations

One major advantage of GWR modeling, which is based on individual locations, is its ability to reveal spatial variations beyond the actual administrative units, and therefore to highlight spatial patterning. Regression parameters can then be seen as new continuous variables, which can lead to a data-based spatial clustering of the relationships for each explanatory variable. As previously shown, GWR also identifies significant associations at the local level that do not appear when fitting the usual global models. Finally, the mapped results are easily readable and can be considered turnkey products for policy-makers. Despite these advantages, GWR has some limitations. First, there are border effects inherent in the concept of spatial kernels. Local regressions for individuals located near the boundary of the study area do not follow exactly the same weighting scheme as those for individuals located in the center, since the former do not have neighbors all around them. This leads to a larger adaptive spatial kernel for these individuals. Second, GWR can lead to local multicollinearity among the explanatory variables, even if the variables are not collinear at a global scale. These facts can affect the validity of the model and the results and therefore require careful consideration.

From a statistical point of view, we performed GWPR models by rounding the outcome variable values to the nearest half-unit (i.e. 30 minutes) in order to get integer values. We also run models using other discretization procedures (rounding to the nearest unit and double unit) to check for statistical stability and findings were essentially unaltered, with unchanged spatial patterning of relationships (data not shown). We also run a geographically weighted logistic regression model by separating active commuters from non-active ones, followed by a second Gaussian GWR model only applied to active commuters, and results again were very similar in terms of direction, intensity and spatial structure of the relationships (data not shown).

In addition, a limitation of our study is the fact that the outcome variable, walking and cycling to/from work, was self-reported. This may be a source of potential misclassification, knowing that physical activity usually tends to be over-reported [18,66,67]. Second, we only focused on active transportation for commuting, but some studies have shown that relationships can be inversed when dealing with walking for leisure or errands. For instance, in four Japanese cities, Inoue et al. [68] showed the expected associations between neighborhood aesthetics and walking in the neighborhood, walking for leisure and walking for daily errands, while no relationship was found with walking for commuting purposes. Finally, this study did not take into account the environmental characteristics of the workplace or the commuting routes, which may also be associated with active transportation behaviors [10,69].

## Conclusion

After showing global, significant associations between individual/environmental factors and active commuting (walking and cycling to/from work) in a French web-cohort, this study implemented a geographically weighted Poisson regression to investigate possible non-stationarity among these associations. At a local scale, GWPR-based analyses enable nuances to be understood by clearly highlighting the spatial heterogeneity of the relationships. For instance, the influence of the overall neighborhood environment appears to be more pronounced in the southern part of the study area (*département* of Val-de-Marne) than in Paris, whereas more complex patterns were revealed elsewhere. We also showed that socio-economic level is significantly and positively associated with the outcome in the extreme northern and southern parts of the area. On the contrary, in some locations, the built environment appears to be non-significantly, or even correlated, in an unexpected way with active commuting (for example in Paris). Perception-based variables are also subject to non-stationarity. Specifically, a better perception of bike safety in traffic is mainly associated with an increase in walking and cycling, except in the northwestern part of the area (Seine-Saint-Denis) where the relationships were inversed. This non-stationarity in the relationships has two main implications. First, from a practical point of view, our results suggest that public policies should follow the spatial patterning of the relationships in order to strengthen their efficiency. GWPR modeling, and its easily readable associated maps, can be a useful tool to guide the design of tailored and area-targeted public policies promoting physical activity for health. Second, from a theoretical point of view, our data suggest that ecological models of health behavior should be not only *population*- and *behavior*-specific but also *location*-specific.

## Appendix A. Global Poisson regression model (GPR)

GPR is a kind of generalized linear model and is typically used for the modeling of count data. The Poisson probability distribution of the number *h* of occurrences of an event is expressed as follows:

$$ P\left(h\Big|\lambda \right)=\frac{e^{-\lambda }{\lambda}^h}{h!}\kern0.75em for\ h=0,1,2, \dots and\ \lambda >0\kern1.5em \left(\mathrm{A}1\right) $$

where *λ* is the only Poisson parameter, as the distribution is equidispersed (i.e. the mean and variance of Poisson distribution are both equal to *λ*_*i*_). In the Poisson regression model, the expected value *λ* is the result of the exponential function of the linear combination of the explanatory variables. Indeed, the log-linear model does not contain negative values of *λ*. Hence, GPR takes the following form:

$$ \log\ {\lambda}_i={\beta}_0+{\beta}_1{x}_{i1}+{\beta}_2{x}_{i2}+\dots +{\beta}_k{x}_{ik}\kern3.9em \left(\mathrm{A}2\right) $$

where *β*_0_, …, *β*_*k*_ are the parameters (or coefficients) of the model and *x*_1_, …, *x*_*k*_ are the predictors (individual and environmental variables). Regression parameters are then estimated by maximizing the log-likelihood in an iterative manner. Because the dependent variable is log-transformed, parameters are interpreted as odds ratios (ORs), just as in logistic regression: each parameter *β*_*i*_ is the estimated increase in the log-odds of the outcome per unit increase in the value of the predictor *x*_*i*_.

## Appendix B. GWPR parameter estimation

According to Nakaya et al. [30], the local parameters can be estimated with a modified local Fisher scoring procedure, a form of iteratively reweighted least squares:

$$ \widehat{\beta}\left({u}_i,{v}_i\right)={\left({\mathbf{X}}^{\hbox{'}}\mathbf{W}\left({u}_i,{v}_i\right)\mathbf{A}\left({u}_i,{v}_i\right)\mathbf{X}\right)}^{-1}{\mathbf{X}}^{\hbox{'}}\mathbf{W}\left({u}_i,{v}_i\right)\mathbf{A}\left({u}_i,{v}_i\right)y\kern5em \left(\mathrm{B}1\right) $$

where *X* is the design matrix of explanatory variables, *W*(*u*_*i*_, *v*_*i*_) is the diagonal spatial weights matrix calculated for each calibration residential location of subject *i*, *A*(*u*_*i*_, *v*_*i*_) denotes the variance weights matrix associated with the Fisher scoring for each residential location of subject *i* and *y* is the *n* × 1 vector of adjusted dependent variables.

## Appendix C. GWPR bi-square weighting scheme

The bi-square function is expressed as follows [28]:

$$ {w}_{ij}=\left\{\begin{array}{c}\hfill {\left(1-{\left({d}_{ij}/{G}_{i(k)}\right)}^2\right)}^2\hfill \\ {}\hfill 0\ \hfill \end{array}\right.\kern0.5em \begin{array}{c}\hfill {d}_{ij}<{G}_{i(k)}\hfill \\ {}\hfill {d}_{ij}>{G}_{i(k)}\hfill \end{array}\kern4.5em \left(\mathrm{C}1\right) $$

where *w*_*ij*_ is the geographical weight of the *j*^th^ observation at the *i*^th^ regression point, *d*_*ij*_ is the Euclidean distance between *i* and *j* and *G*_*i*(*k*)_ is the adaptive bandwidth size defined as the *k*^th^ nearest neighbor distance.
